# Lessons Learned from Approval of Aducanumab for Alzheimer’s Disease

**DOI:** 10.1146/annurev-med-051022-043645

**Published:** 2024-01-29

**Authors:** Judith L. Heidebrink, Henry L. Paulson

**Affiliations:** 1 Department of Neurology, University of Michigan, Ann Arbor, Michigan, USA;; 2 Michigan Alzheimer’s Disease Center, University of Michigan, Ann Arbor, Michigan, USA; 3 Michigan Neuroscience Institute, University of Michigan, Ann Arbor, Michigan, USA

**Keywords:** dementia, antiamyloid, immunotherapy, drug approval, clinical trials, accelerated approval

## Abstract

When the US Food and Drug Administration used the accelerated approval process to authorize the use of the antiamyloid drug aducanumab to treat Alzheimer’s disease (AD), many people hoped this signaled a new era of disease-modifying treatment. But 2 years later, aducanumab’s failure to launch provides a cautionary tale about the complexities of dementia and the need for a thorough and transparent review of the role that regulatory agencies and various stakeholders play in approving AD drugs. We highlight the events leading to aducanumab’s controversial approval and discuss some of the key lessons learned from the drug’s failure to deliver the hoped-for benefits. These lessons include the inherent limitations of antiamyloid strategies for a complex disease in which amyloid is only one of several pathological processes, the need for clinical trials that better reflect the diversity of communities affected by AD, the potential pitfalls of futility analyses in clinical trials, the need for greater transparency and other modifications to the approval process, and the dementia field’s unreadiness to move from the highly controlled environment of clinical trials to the widespread and chronic use of resource-intensive, disease-modifying drugs in real-world treatment scenarios. People with dementia desperately need effective therapies. We hope that the aducanumab story will inspire changes to the approval process—changes that restore public trust and improve future efforts to deliver disease-modifying therapies to the clinic.

## RISE AND FALL OF ADUCANUMAB

On June 7, 2021, the US Food and Drug Administration (FDA) granted accelerated approval to aducanumab, a monoclonal antibody targeting beta-amyloid, for the treatment of Alzheimer’s disease (AD). Hailed as the first disease-modifying therapy for AD and the first AD drug approved since 2003, aducanumab was initially projected to receive widespread adoption, with estimated yearly sales in the billions based on a $56,000/year list price (1). Soon after aducanumab’s approval, its maker Biogen launched the International Collaboration for Real-World Evidence in Alzheimer’s Disease (ICARE AD) postmarketing study. ICARE AD was designed to evaluate the real-world safety and effectiveness of aducanumab in US clinical practice. However, ICARE AD enrollment and overall aducanumab prescribing fell far short of initial projections. In December 2021, Biogen announced a price cut of nearly 50% in hopes of increasing patient access (2). The Centers for Medicare & Medicaid Services (CMS) issued a national coverage decision (NCD) on aducanumab and similar antibodies in April 2022 (3). This NCD restricts coverage for antibodies receiving accelerated approval to FDA-approved randomized controlled trials or studies supported by the National Institutes of Health. In response to the NCD, ICARE AD was terminated in May 2022, given the expectation of limited aducanumab use in clinical practice (NCT05097131). At the time of termination, 29 participants were enrolled.

Why did aducanumab fall from potential blockbuster status to being all but dead commercially? Was there a shift in scientific or public opinion following its FDA approval, or was it ill-fated from the start? More importantly, what can scientists and healthcare professionals learn from the rise and fall of aducanumab? Here we review aducanumab’s controversial path to approval, its failure to launch as a potential disease-modifying therapy, and key lessons for the future of AD treatment.

### Amyloid Cascade Hypothesis

**Figure 1:**
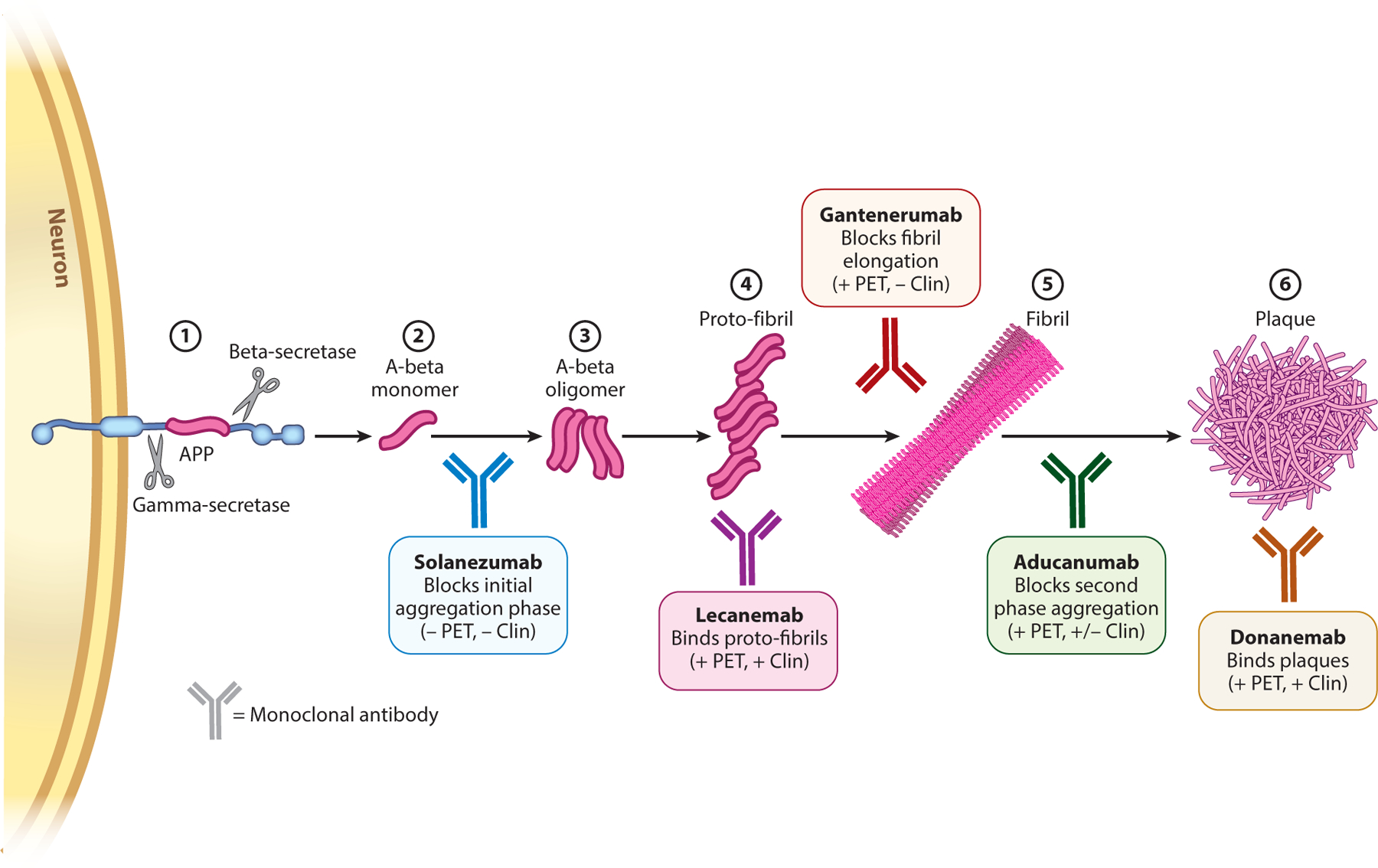
The amyloid cascade and antiamyloid immunotherapy. The amyloid cascade begins with (①) proteolytic cleavage of the amyloid precursor protein (APP) at the cell surface of neurons, (②) generating the aggregation-prone beta-amyloid peptide (A-beta). (③) A-beta monomer readily oligomerizes, then forms (④) proto-fibrils, which mature into highly ordered (⑤) fibrils that (⑥) accumulate in amyloid plaques. Shown are aducanumab and four other monoclonal antibodies that recently underwent or are still undergoing testing in clinical trials. The various points along the amyloid cascade at which these specific antibodies are thought to act are highlighted (two other tested antibodies, bapineuzimab and crenezumab, act at multiple points along the cascade and are not shown). Antibodies that effectively reduce brain amyloid based on positron emission tomography (PET) imaging are indicated by + PET. The presence or absence of a clinical effect is indicated by + Clin or − Clin, respectively.

A discussion of aducanumab first requires a review of the role of amyloid in AD. The most common cause of dementia in older adults, AD is characterized pathologically by the accumulation of extracellular beta-amyloid plaques and intracellular tau tangles. Beta-amyloid arises from the sequential cleavage of the amyloid precursor protein (APP) by two proteases, beta- and gamma-secretase. The resulting beta-amyloid peptide, only 40–42 amino acids in length, is soluble but prone to aggregation in increasingly larger forms: monomers, oligomers, proto-fibrils, fibrils, and plaques (Figure 1). Amyloid is the general term for highly ordered fibrillar structures formed by various proteins in many disease states, which in the case of AD plaques are formed specifically from beta-amyloid peptides.

The amyloid cascade hypothesis posits that beta-amyloid aggregation initiates and drives AD pathogenesis, with tau-mediated toxicity occurring subsequently (4). Several lines of evidence support this hypothesis, including the discovery of causal mutations in APP and in the two presenilin subunits of the gamma-secretase complex, all of which result in overproduction of beta-amyloid (5). Accordingly, much of AD drug development has focused on therapies targeting beta-amyloid. Chief among these therapies thus far has been antiamyloid immunotherapy.

### Immunotherapy

Immunotherapy can be (*a*) active, administering a vaccine containing beta-amyloid or its fragments to induce a humoral response, or (*b*) passive, infusing beta-amyloid antibodies. In mouse models of AD, both active and passive immunotherapies have cleared brain amyloid (6, 7). However, early vaccine trials in humans were limited by unpredictable immune responses. In a phase IIa trial of AN1792 in persons with mild–moderate AD dementia, only 20% generated the prespecified level of antibody titers (8). Of greater concern, 6% of treated participants developed a T cell–mediated meningoencephalitis, resulting in study termination.

Attention shifted to passive immunotherapy, in which antibodies are delivered at controlled doses and times. Over the past decade, numerous monoclonal antibodies have been studied in individuals with symptomatic AD (Table 1) (9–16). While all target beta-amyloid, they differ in their binding epitopes and their selectivity for soluble versus oligomeric versus aggregated forms of the disease-linked protein (Figure 1).

### Aducanumab: Amyloid Effect

Aducanumab is a human monoclonal antibody derived from the lymphocytes of older blood donors who showed no or minimal cognitive decline with age (17). Preclinical studies demonstrated selectivity for aggregated forms of beta-amyloid and low affinity for soluble monomers (17, 18). In a transgenic mouse model, parenchymal plaques of all sizes were reduced after 6 months of weekly dosing, but vascular amyloid was unchanged (18).

Aducanumab’s effect on brain amyloid in humans was first studied in the phase Ib PRIME trial (18). PRIME enrolled 165 participants with early symptomatic AD: mild cognitive impairment (MCI) or mild dementia thought to be due to AD, and a positron emission tomography (PET) scan positive for brain amyloid. Participants received infusions every 4 weeks of either placebo or aducanumab at 1, 3, 6, or 10 mg/kg. Repeat PET scans after 1 year showed a marked, dose-dependent reduction in brain amyloid. Scans in those receiving the highest dose showed a drop in amyloid to near the threshold of positivity, effectively clearing deposited amyloid from the brain.

PRIME also demonstrated a dose-dependent side effect known as ARIA (amyloid-related imaging abnormalities). There are two types of ARIA: (*a*) ARIA-E (edema), characterized by magnetic resonance imaging (MRI) evidence of vasogenic edema and sulcal effusions; and (*b*) ARIA-H (hemorrhage), characterized by MRI evidence of hemosiderin deposits. ARIA-E was more common with increasing aducanumab dose (3%, 6%, 37%, and 41% for 1, 3, 6, and 10 mg/kg, respectively) and in individuals harboring the apolipoprotein E4 (apoE4) allele (55% versus 17% for carriers versus noncarriers at 10 mg/kg). In most participants, ARIA-E was asymptomatic, but approximately one-third had symptoms such as headache, visual disturbances, or confusion that typically resolved within 4 weeks. Across aducanumab groups, 44% of those who developed ARIA-E discontinued treatment. No participants were hospitalized due to ARIA, and there were no drug-related deaths.

### Aducanumab: Clinical Effect?

Biogen launched two phase III trials of aducanumab, EMERGE and ENGAGE (13). Inclusion criteria were identical and, like PRIME, these trials focused on early symptomatic AD. Each study had three arms: placebo, low-dose aducanumab, or high-dose aducanumab infusions every 4 weeks. Given concerns about ARIA in apoE4 carriers, the trials initially used different low (3 mg/kg versus 6 mg/kg) and high (6 mg versus 10 mg/kg) doses for apoE4 carriers versus noncarriers. Protocol revisions later allowed apoE4 carriers to receive the same dose as noncarriers within a given arm.

EMERGE and ENGAGE were originally intended as 18-month trials. However, in March 2019, Biogen terminated both trials early based on an interim futility analysis that predicted aducanumab would be ineffective. In a surprising reversal, Biogen announced 6 months later that their prediction was wrong and that the EMERGE high-dose group had met its primary endpoint. The primary outcome measure was the clinical dementia rating (CDR), a semistructured interview of the participant and an informant (19). For the CDR, a clinician assigns a rating in each of six areas (memory, orientation, judgment, community affairs, home and hobbies, and personal care) according to the degree of impairment in that area: 0 (none), 0.5 (very mild), 1 (mild), 2 (moderate), or 3 (severe). In EMERGE, the CDR-Sum of Boxes (total score for all 6 areas) increased by 1.74 in the placebo group and by 1.35 in the high-dose group, for a difference of 0.39 or a 22% slowing of decline (*p* = 0.01). Secondary outcome measurements in EMERGE also indicated less progression in the high-dose group. In contrast, there was no statistically significant difference in any primary or secondary outcome in the ENGAGE trial. Across both studies, ARIA-E was the most common adverse event; it occurred in 35% of the high-dose group overall, and more often in apoE4 carriers (65% in homozygous carriers, 35% in heterozygous carriers, and 20% in noncarriers) (13, 20). In the high-dose group, about 25% of ARIA-E was symptomatic, with a median symptom duration of 5 weeks. Headache was the most common symptom. Most ARIA-E occurred within the first 8 infusions. If ARIA was radiographically mild and asymptomatic, treatment continued. Treatment was suspended or discontinued if ARIA was of greater radiographic severity or symptomatic. Severe ARIA symptoms with aducanumab were uncommon and included headache (four patients), confusion, seizure, and weakness due to cerebral hemorrhage (one patient each).

### FDA Review

Following the reanalysis of EMERGE and ENGAGE, Biogen applied for FDA priority review of aducanumab. In November 2020, an external FDA advisory committee overwhelmingly recommended against approval, finding insufficient evidence of clinical effectiveness (21). At the meeting, committee members were not asked to consider whether reduction in amyloid PET signal met criteria for a surrogate endpoint (21). The FDA postponed its decision until June 2021 after requesting additional information from Biogen. On June 7, 2021, the FDA approved aducanumab via the accelerated approval pathway. This pathway allows approval of drugs for the treatment of serious diseases with unmet needs based on a surrogate endpoint. In this case, the surrogate endpoint was reduction of beta-amyloid plaque in the brain as measured by PET. The FDA required a confirmatory trial, to be completed within 9 years, to verify aducanumab’s anticipated clinical effect.

The initial aducanumab label granted approval “for the treatment of Alzheimer’s disease” without regard to severity (22). The label was amended a month later to limit use to those with the MCI or mild dementia stage of AD (23). However, it did not specify the need for an amyloid biomarker, leaving concerns about potential inappropriate use (21).

By September 2021, three members of the external advisory committee had resigned, with some citing a process that “made a mockery” of the advisory committee’s role and resulting in “what might be the worst approval decision that the FDA has made that I can remember” (24). Concerns about inappropriate interactions between Biogen and the FDA led to an 18-month congressional investigation, culminating in a December 2022 report highly critical of the approval process (25). The findings included an approval process “rife with irregularities,” such as an “atypical” degree of collaboration between Biogen and the FDA and numerous interactions in which the “FDA failed to follow its own documentation protocol” (25, p. 7). In addition, the FDA was faulted for issuing a broad label and for dismissing its own statistical division’s unfavorable review of the aducanumab data (25). The congressional committees decried Biogen’s $56,000 pricing strategy designed to achieve a blockbuster launch. In comparison, the Institute for Clinical and Economic Review (ICER) estimated a cost-effective price of $3,000–$8,400 per year, assuming aducanumab produced a net health benefit, for which ICER felt there was insufficient evidence (26).

### Approved But Unused

In the aftermath of the FDA’s controversial approval, health systems such as the Cleveland Clinic, Mount Sinai Health System, Mass General Brigham, University of Michigan, and the Department of Veterans Affairs declined to offer aducanumab based on safety and efficacy concerns (27). The Alzheimer’s Association campaigned heavily for patient access to aducanumab and advocated for a price reduction and CMS coverage (28). Medicare Part B monthly premiums for 2022 rose by $21.60 in anticipation of the cost of covering aducanumab, only to fall by $5.20 for 2023 following CMS’s decision to restrict aducanumab coverage to qualified studies (29).

CMS issued its final NCD following a 30-day comment period, during which it received 131 comments. The majority (~77) did not support coverage or advised coverage with further evidence development, and 26 did not express a clear position on coverage (3).

### Reflections

Up to the time of aducanumab’s approval, there had been no effective disease-modifying therapy for the millions of Americans affected by AD, despite decades of effort by an increasingly large clinical research enterprise and extensive development efforts of many pharmaceutical companies (30). The accelerated FDA approval of aducanumab was initially viewed by advocacy groups and many patients as a ray of hope, because it appeared to signal a new era of treatment that could relieve the despair and desperation experienced by so many families afflicted with AD. The failure of aducanumab to launch as a disease-modifying drug instead provides a cautionary tale regarding the complexities of AD and the importance of thorough and transparent review by regulatory agencies when designating approval for AD drug treatments in the future. Below, we highlight some of the key lessons learned from this still-evolving story.

## LESSONS LEARNED

### Amyloid Immunotherapy May Be a Hit, But Not a Home Run

The early termination of EMERGE and ENGAGE and their disparate outcomes obscured a clear picture of aducanumab’s clinical effect, or lack thereof, in early AD. Nonetheless, even the most optimistic interpretation of the data is that aducanumab slows progression slightly but does not halt or reverse it. In EMERGE, after 18 months the difference in CDR increase between high-dose aducanumab and placebo was 0.39—less than the minimum possible difference between two CDR scores (0.5). Although there is no expert consensus on what constitutes a clinically meaningful difference on the CDR, a full 1-point increase over 1 year has been suggested as the threshold that would indicate meaningful decline for someone with MCI (31, 32).

How does aducanumab compare to other monoclonal antibodies? Phase III trials of bapineuzumab, solanezumab, crenezumab, and gantenerumab in symptomatic AD also failed to meet their primary endpoints (see Table 1). Lecanemab, which received FDA approval on July 6, 2023, met all its primary and secondary endpoints in a large phase III trial called CLARITY AD (14). For lecanemab, the CDR difference was 0.45, representing a 27% slowing of decline, or approximately 6 months less decline over the 18-month trial. Slowing was similar (32%) in a phase II trial of donanemab (16).

ENGAGE and gantenerumab’s GRADUATE trials were negative despite end-of-study measurements of brain amyloid reduction that revealed an effect similar to that seen in EMERGE and with lecanemab (15). The poor correlation between the reduction in amyloid PET signal and clinical treatment response casts doubt on the use of brain amyloid PET results as a surrogate marker for clinical efficacy. However, as argued by Karran & De Strooper (33), dramatic brain amyloid reduction, equivalent to “normalizing” an amyloid PET scan, may be necessary to prevent downstream tau pathology, and there may well be a significant time lag between amyloid reduction and observable clinical benefit. Thus, a therapy that rapidly and effectively lowers amyloid combined with a longer period of observation might best determine the full potential of amyloid immunotherapy.

Any “time savings,” such as the 6 months less decline with lecanemab, must be weighed against the risk of side effects such as ARIA. If ARIA primarily occurs early but the clinical benefit grows over time, then consensus likely will eventually be achieved that immunotherapy has clinically meaningful benefits. However, establishing this requires longer trials, as advised by the authors of CLARITY AD (14).

While amyloid deposition appears necessary for AD, it is not sufficient. Indeed, the pattern of tau accumulation tracks more closely with clinical features of disease and brain atrophy than does plaque distribution (34, 35). Current hypotheses support models in which AD is a beta-amyloid initiated tauopathy in which immune cells, including microglia, are important mediators (36). Older individuals demonstrating resilience (continued normal cognition despite amyloid accumulation) are of great research interest because they can provide clues to disease-relevant pathways and therapeutic targets beyond amyloid (37–39). The future of disease-modifying therapy in AD may include antiamyloid drugs, but most likely additional therapies targeting tau and immune pathways will be required for robust clinical efficacy (33, 40). As cancer and HIV disease illustrate, multi-pronged therapy is often required for effective treatment, and the chronic and complex nature of AD likely will require a multi-targeted approach. In the push for disease-modifying therapies, it would be shortsighted to neglect further studies of symptomatic therapies, including studies that combine symptomatic and potential disease-modifying treatment strategies (41).

### Trials Should Capture the Diversity of Those with AD

CMS announced in its NCD that monoclonal antibodies receiving full FDA approval may be covered in CMS-approved prospective comparative studies (3). These studies must include “a study population whose diversity of patients [is] representative of the national population with MCI due to AD or mild AD dementia.” Despite disproportionately higher rates of AD among Hispanic/Latino and Black/African American populations (42), these groups were under-represented in EMERGE/ENGAGE (only 3% and <1%, respectively). Many societal, environmental, and genetic factors contribute to the varied manifestations of AD across diverse populations. Effective clinical trials must enroll a diverse range of participants in order to mirror society and determine clinical effects in different populations.

Requiring greater diversity among patients enrolled in AD trials also may serve as a stepping-stone to ensuring that any identified effective drug becomes available to the study populations in which it is found effective. Significant health disparities exist in the diagnosis and treatment of AD and related dementias, disproportionately affecting Hispanic/Latino and Black/African American communities (43). A community’s trust in new therapies is more likely to be gained when that community has been engaged in studies establishing the drug’s efficacy; fortunately, concerted efforts are underway to bring AD clinical trials to more diverse communities, including historically excluded groups (44–46).

### Futility Analyses Come with Risks

The aducanumab story points to the challenges and potential pitfalls of futility analyses (47). Ethically, a trial must be stopped early if the risk/benefit ratio becomes unfavorable due to unexpected adverse events. This was the case with the earlier AN1792 amyloid vaccine trial (8). In contrast, stopping a trial early for futility can be ethically problematic. A common justification is to avoid spending resources on an ineffective therapy. Yet, resource expenditure is disproportionately greater at the beginning of a trial due to start-up processes, including recruitment. Thus, a mid-point futility analysis leading to early termination would not save half the study resources. Of greater concern, a futility analysis can wrongly estimate the true treatment effect, depending on its assumptions. Such was the case with EMERGE and ENGAGE. The futility analysis wrongly assumed a similar treatment effect in both studies and a constant treatment effect over time (13). These assumptions were not revisited despite protocol amendments that produced different dose exposures over time within and between the two studies.

Early, abrupt termination can also have devastating effects on trial participants, particularly if they are unaware of the possibility of early stoppage and learn of it first through national news outlets rather than from their local study team (48). In response to such experiences with aducanumab and other trials halted early in 2018–2019, the Participant Follow-Up Improvement in Research Studies and Trials (Participant FIRST) Work Group was formed (49). Participant FIRST issued 17 key recommendations to improve communication and support participants and their study partners when trials end early. These recommendations include addressing the possibility of early stoppage during the consent process, rapidly notifying participants by email and by phone when there is early stoppage, and having a personalized close-out meeting.

### Restoring Trust Is Critical

Confidence of Americans in medical scientists has eroded over the past few years, according to a 2022 Pew Research Center survey (50). While American support for dementia science is strong, the missteps of the aducanumab story threaten public trust in dementia research (51, 52). Accordingly, the various entities engaged in AD research and drug development—scientists, clinicians, pharmaceutical companies, advocacy groups, and regulatory agencies—must work together in a transparent, deliberative, and dispassionate manner to ensure we do not lose sight of the ultimate goal, which is to identify and deliver effective treatment for those suffering from cognitive impairment.

The passionate dedication of advocacy groups like the Alzheimer’s Association has greatly accelerated dementia-related research and the search for therapies. We commend these groups for being such a strong voice for those confronting dementia. The passionate search for treatments, however, needs to be responsive to the scientific facts. The push to approve aducanumab was well-intentioned, but it led regulators to disregard certain important procedures and to give too little weight to the assessments of expert scientists, including, most importantly, the FDA advisory committee. New measures should be adopted to ensure that the norms of regulatory science are not ignored in the quest for effective treatment in AD. This particularly applies to the accelerated approval pathway. Following the aducanumab approval process, Largent and colleagues proposed that the FDA adopt procedural changes to promote “reasonableness” in accelerated approval decision-making (53). Domains of reasonableness raised by the authors include “pathway gatekeeping,” which would require sponsors to declare, at the time of trial design, their intention to seek accelerated approval; “endpoint selection,” in which the FDA would transparently evaluate a potential surrogate endpoint before endorsing its use; “stakeholder engagement” efforts, through which the FDA would engage and seek the opinions of a larger and more diverse group of patients, among other stakeholders; and greater “deliberativeness” to ensure that all angles in an approval decision are considered and promote neutrality in the FDA’s decision-making process.

The approval process of a disease-modifying drug that would likely be taken chronically by millions of Americans requires a high level of scrutiny to ensure safety and efficacy. Data relied on to drive the approval process should be broadly accessible to the scientific community in a timely manner. The aducanumab trials, for example, were not published until well after FDA review. It is also important that there be fully transparent communication between companies and the FDA and its advisory groups. Greater transparency likely will bolster public trust in the process and diminish the likelihood that external pressures that are not germane to ensuring safety and efficacy will intervene in the approval process. It will also aid the FDA in properly defining the range of coverage (restricted versus broader patient populations), contraindications to treatment, and appropriate safety monitoring measures.

Finally, the scientific community and advocacy groups should redouble efforts to ensure that the American public is well informed about advances in dementia science and treatment. A recent survey showed that most of the US public was unaware of aducanumab (54). But when provided information about the approval process, survey participants expressed concern about the controversial pathway, supported measures to restrict drug use to those patients most likely to benefit, and were willing to participate in further randomized placebo-controlled trials. The opinions of an informed public, the authors concluded, should be considered when developing policies in response to aducanumab’s approval.

### We Are Unprepared for Real-World Treatment

There are many practical challenges to delivering even the most promising AD immunotherapy outside of a clinical trial. Patients need access to dementia specialists who can determine their eligibility and monitor their safety during treatment. Currently, however, most individuals with dementia do not see a specialist within 5 years of their diagnosis (55). The availability of immunotherapy creates an even greater demand for specialty care. In a simulation analysis of US preparedness to provide a therapy such as aducanumab, the availability of dementia specialists was identified as the most pressing constraint (56). Formal neuropsychological testing to recognize the presence of MCI is also often a limited resource in many healthcare settings.

Antiamyloid immunotherapy requires evidence of amyloid pathology. Clinical trials typically use amyloid PET imaging, but PET scanners and amyloid tracers are not widely available geographically. Measurement of amyloid in cerebrospinal fluid is an alternative but necessitates a lumbar puncture as well as special handling and processing of samples (57). MRI must be available (*a*) at baseline to assess for any contraindications, (*b*) at recommended intervals during treatment to assess for ARIA, and (*c*) on an unscheduled basis should symptoms suggesting ARIA emerge. Since rates of ARIA vary with apoE4 status, apoE testing is advised, adding to the detailed discussion between provider, patient, and care partners to determine appropriateness of treatment. For aducanumab, provider time to review and discuss eligibility was estimated to be 1.5–2 h per patient, with considerably more time required of support staff to coordinate screening studies, insurance authorization, and infusions (58).

Finite resources coupled with patient demand may lead to treatment of individuals who would not have met trial eligibility criteria, potentially altering the risk/benefit ratio. Even when individuals match trial criteria, real-world treatment will not entail the same monitoring as occurs in a clinical trial. For example, trial MRIs are reviewed by central radiologists with expertise in ARIA, whereas a local radiologist may have no experience in detecting ARIA.

Finally, in addition to resource limitations and safety considerations, there are unanswered questions when moving from clinical trials to real-world use. Beyond holding/stopping treatment, how should symptomatic ARIA be managed? How long should treatment continue? Should treatment duration be guided by amyloid biomarkers?

## SUMMARY

This review had two goals: first, to chronicle the steps leading to the approval and eventual failure of aducanumab; and second, to highlight areas where procedures can be improved so that this unfortunate experience is not repeated. Chronicling how it happened constitutes the first step toward implementing necessary change. The dementia field needs to know where procedural deficiencies reside so that we can collectively push for reform. Achieving this needed reform will help to restore public trust in the scientific process and, eventually, bring us the disease-modifying treatments we have long sought. Here, we have not offered solutions to the problem. We are, however, encouraged by the efforts already underway by various stakeholders to enact regulatory and procedural changes. Soon, we hope, the “lessons learned” outlined here will be followed by “changes enacted” that lead to successful dementia drug approval and implementation.

## Figures and Tables

**Table 1:** Monoclonal antibody trials in symptomatic Alzheimer’s disease

Monoclonal antibody	AD stage	*n*	Planned length (years)	Primary outcome(s)	Clinical effect	Amyloid PET change	Reference
Bapineuzumab	Mild/mod	2,204	1.5	ADAS-11, DAD	NS	NS	9
Solanezumab	Mild/mod	2,052	1.5	ADAS-11, ADL	NS	NS	10
Solanezumab	Mild	2,129	1.5	ADAS-14	NS	NS	11
Crenezumab	Early	1,619	2	CDR-SB	NS	NS	12
Aducanumab	Early	1,647	1.5	CDR-SB	NS	−54 Centiloids (59% decrease)	13
Aducanumab	Early	1,638	1.5	CDR-SB	0.39 Less increase (22% slowing)	−61 Centiloids (71% decrease)	13
Lecanemab	Early	1,795	1.5	CDR-SB	0.45 Less increase (27% slowing)	−55 Centiloids (71% decrease)	14
Gantenerumab	Early	1,965	2	CDR-SB	NS	−53 Centiloids (56% decrease)	15
Donanemab^a^	Early	1,736 Combined tau levels 1,182 Low/medium tau level	1.5	iADRS	2.92 Less decrease (22% slowing) 3.25 Less decrease (35% slowing)	−87 Centiloids (84% decrease) −88 Centiloids (86% decrease)	16

a The donanemab trial enrolled participants with either low/medium or high tau as measured by PET. Low/medium tau was the primary analysis population.

Abbreviations: AD, Alzheimer’s disease; ADAS-11 or ADAS-14, Alzheimer’s Disease Assessment Scale–Cognitive Subscale (11 or 14 items); ADL, Alzheimer’s Disease Cooperative Study–Activities of Daily Living scale; CDR-SB, Clinical Dementia Rating scale Sum of Boxes; DAD, Disability Assessment for Dementia; early, mild cognitive impairment or mild dementia; iADRS, Integrated Alzheimer’s Disease Rating Scale; mild/mod, mild or moderate dementia; NS, not significant; PET, positron emission tomography.

## Abbreviations

CMS: Centers for Medicare & Medicaid Services
NCD: national coverage decision
MCI: mild cognitive impairment
ARIA: amyloid-related imaging abnormalities
